# The barriers to cervical cancer screening for urban and rural populations in Rwanda

**DOI:** 10.1186/s44263-023-00005-6

**Published:** 2023-07-31

**Authors:** Hallie Dau, Marianne Vidler, Maryam AboMoslim, Barbra Mutamba, Zoey Nesbitt, John Deodatha, Schadrack Danson Byiringiro, Charles Niyotwiringiye, Nadia Mithani, Varun Nair, Laurie Smith, Stephen Rulisa, Gina Ogilvie

**Affiliations:** 1 School of Population and Public Health, University of British Columbia, Vancouver, BC Canada; 2 Women’s Health Research Institute, Vancouver, BC Canada; 3 Department of Obstetrics and Gynaecology, University of British Columbia, Vancouver, BC Canada; 4 Eagle Research Center, Kigali, Rwanda; 5 Integrated Sciences, University of British Columbia Vancouver, British Columbia, Canada; 6 BC Cancer, Vancouver, Canada; 7 School of Medicine and Pharmacy, University Teaching Hospital of Kigali, University of Rwanda, Kigali, Rwanda; 8 BC Centre for Disease Control, Vancouver, Canada; 9 BC Women’s Hospital and Health Centre, Box 42, Room H203G - 4500 Oak Street, Vancouver, BC V6H 3N1 Canada

**Keywords:** Rwanda, Cervical cancer, Screening, Barriers

## Abstract

**Background:**

Cervical cancer is the leading cause of cancer mortality in Rwandan women. There is a limited understanding of the barriers that women face to obtain cervical cancer screening in Rwanda. It is important to understand the barriers in order to implement effective screening programs. The goal of this study is to describe the barriers to cervical cancer screening among women in Rwanda and how they differ among women in rural and urban areas.

**Methods:**

This cross-sectional study recruited women from June 1 to 9, 2022, at Muhima and Nyamata District Hospitals in Rwanda. Women were eligible for the study if they were ≥ 18 years and spoke Kinyarwanda or English. Women completed a 15-min survey which included questions on the participants’ demographics, knowledge of cervical cancer, cervical cancer screening history, and barriers to healthcare. Women were stratified by survey location (urban vs rural). Descriptive statistics were reported.

**Results:**

A total of 374 women completed the survey with 169 participants from Muhima and 205 from Nyamata. Most women were in a relationship and had a primary school or less education. The most common barriers to accessing general healthcare services were long wait times at the facility (Muhima 26%; Nyamata 30%), low quality of care (Muhima 15%; Nyamata 12%), and transportation costs (Muhima 13%; Nyamata 9.3%). However, women from Nyamata were significantly more likely to report distance to the health center as a barrier (*p*-value < 0.001), and women from Muhima were significantly more likely to report transportation method as a barrier (*p*-value = 0.004). The primary reason reported for not obtaining cervical cancer screening was that women did not know how or where to get tested (Muhima 57%; Nyamata 51%).

**Conclusions:**

The most common barriers to cervical cancer screening in Rwanda were the quality of clinical care and issues with traveling to the clinic. Implementing a cervical cancer self-collection program could help eliminate many barriers that women face to obtain health services in Rwanda. More research is needed to better understand the acceptability of cervical cancer screening in Rwanda and how it could be integrated into the healthcare system.

## Background

Cervical cancer is one of the most preventable types of cancer because of vaccination and effective widespread screening methods. However, globally it remains the fourth most common type of cancer among women [1]. In low- and middle-income countries (LMIC), it has one of the highest incidence and mortality rates among female cancers [2] with sub-Saharan Africa (SSA) carrying the highest burden due to poor infrastructure and financial constraints [1, 3]. The World Health Organization has called for the elimination of cervical cancer, with the ambitious goal of achieving 90–70-90, where 90% of girls are fully vaccinated with the HPV vaccine by 15 years of age, 70% of women are screened at least twice by age 45, and 90% of women with pre-cancer or cancer receive the appropriate care and treatment [4]. While the HPV vaccine is highly effective in reducing the risk of cervical cancer, its affordability and unequal distribution have led to less than 25% of low-income countries administering it as part of their immunization programs [4]. Therefore, screening and treatment of cervical cancer and pre-cancer must be of top priority if cervical cancer is to be eliminated.

The cervical cancer vaccination program in Rwanda has been widely successful. Rwanda was the first African country to implement a national vaccination program against HPV in 2011 [5]. In 2012, the program reached 96.6% coverage of the target population after the catch-up period. As a result, Rwanda has one of the highest HPV vaccination rates in the world [6]. However, while the vaccination program achieved widespread coverage among young girls, challenges remain with cervical cancer screening. Cervical cancer is the leading cause of cancer deaths in Rwandan women [1]. As the vaccine program has only been implemented for approximately 10 years and is offered to girls in grade six [5], many women in Rwanda never received the vaccine as they were too old when the program was initiated [7]. This, in turn, puts them at a higher risk for developing cervical cancer. There is a need to better understand the barriers that women face in Rwanda to access cervical cancer screening in order to implement an efficient program.

Generally, the barriers to cervical cancer screening in LMICs are well known. Commonly reported barriers include knowledge of screening methods [8–10], cost [8, 11], shame [8], awareness of the importance of screening [11], and access to health facilities [9, 10]. While understanding barriers in LMICs as a whole is important, it is also important to consider the country-specific barriers. To date, only one peer-reviewed quantitative study has examined barriers in Rwanda. Niyonsenga et al. published a cross-sectional study in 2021 and reported that the most common barriers were knowledge of availability, lack of awareness, and living in rural areas [12]. However, this study only sampled women from three district hospitals in urban Kigali.

The overall goal of understanding the barriers to cervical cancer screening is part of a larger aim of this study to determine if self-collection would be an acceptable method for cervical cancer screening in Rwanda. Self-collection for cervical cancer screening has proven to be an accurate low-cost method of HPV testing when compared to other methods such as visual inspection with acetic acid (VIA) [13–15]. Importantly, it has also shown to be highly acceptable among women [16]. A 2010 study by Mitchell et al. in Uganda found that over 80% of women indicated that they were willing to self-collect cervical samples [16]. While the willingness to self-collect has been studied in multiple countries [16–19], there is no knowledge if self-collection would eliminate the barriers to cervical cancer screening in Rwanda. As such, this study aims to describe the perceived barriers to cervical cancer screening among women in Rwanda and how they differ among women in rural and urban areas.

## Methods

### Data collection

This cross-sectional study surveyed women waiting for health services at Muhima and Nyamata District Hospitals in Rwanda. Muhima and Nyamata districts were chosen as they represent both urban and rural districts in Rwanda. Muhima is an urban hospital located in Kigali and Nyamata is a rural hospital located in the Bugesera district. Each hospital was provided with 150,000 Rwanda Franc ($140 USD) by the study team as a token of appreciation for their participation in the study. Women were eligible for the study if they (i) were over the age of 18 years, (ii) spoke Kinyarwanda or English, and (iii) were able to provide informed consent. Women were recruited from June 1 to 9, 2022. Participants were recruited from the waiting room by four Rwandan data collectors fluent in both Kinyarwanda and English using convenience sampling. The survey was administered on tablets using REDCap software [20]. All self-reported questions were read aloud to participants and entered into REDCap by the data collectors in real time. Participants were compensated 1050 Rwanda Franc ($1 USD) for participating in the study.

### Measures and analysis

The survey tool consisted of 51 closed-ended questions which took participants approximately 15 min to complete. The survey included questions on the participants’ demographics, knowledge of cervical cancer, cervical cancer screening history, barriers to screening, integration of cervical cancer screening services, and willingness to self-collect for cervical cancer screening. The survey tool is a combination of two survey instruments: (i) the core plus module of the Improving Data for Decision Making in Global Cervical Cancer Programs Toolkit-Part 2 (IDCCP) [21] and (ii) a survey conducted in Kisenyi, Uganda, by Mitchell et al. [16] which is informed by the Theory of Planned Behaviour [22], comprehensive literature reviews, and expert interviews. Module 1 of the IDCCP toolkit includes core questions on screening prevalence, interval, results, and treatment and 14 optional questions.

Perceived barriers to cervical cancer screening were measured using the following survey items. The first item asked women what their biggest challenge was accessing women’s health services. Response options included long wait times at the facility, low quality of care, transportation cost, distance to health center, health care workers not receptive, transportation method, lack of awareness of where to get services, no time, partner not supportive, lack of awareness on what services I need, other, not important, or none. The second item asked women what the primary reason was as to why they have never received a cervical cancer screening test. Response options included poor service quality, clinic too far away, family member would not allow it, embarrassment, afraid of the procedure, did not have time, did not know how/where to get the test, other, and do not know.

Participants’ demographics were also included in the analysis; this included survey location, marital status, education, religion, age at the first time they had sexual intercourse, number of sexual partners in the last week, comorbidities, number of visits to a health facility in the past 12 months for a reason other than pregnancy, if they had ever been screened for cervical cancer, the most commonly accessed services at a health facility, and willingness to self-collect at their home for cervical cancer screening. Before answering questions about self-collection, data collectors were instructed to read a script on self-collection procedures that included a demonstration. Willingness to self-collect included five response options which were dichotomized into yes–no. The yes category included somewhat likely and very likely. The no category included not sure, somewhat likely, and very unlikely.

All descriptive statistics were calculated using counts and frequencies. Chi-square and Fisher’s exact tests with complete cases were used to compare barriers to self-collection between women who completed the survey in Muhima and Nyamata. *P*-values were used to report all significant levels with < 0.05 indicating a significant difference between the two groups. Missing values were provided for all variables that contained missing data. All analyses were conducted using R 4.2.3 [23].

## Results

The survey was completed by 374 participants in Kinyarwanda (Table 1). No participant completed the survey in English. In all, 169 (44.2%) participants completed the survey in Muhima and 205 (54.8%) in Nyamata. Most women were in a relationship (Muhima *n* = 123, 73%; Nyamata* n* = 164, 80%; *p*-value 0.082) and had a primary school or less education (Muhima *n* = 84, 51%; Nyamata *n* = 132, 65%; *p*-value 0.009). The majority of women in both groups had made three or more visits to a health facility in the past 12 months for reasons other than antenatal care (ANC) (Muhima *n* = 82, 51%; Nyamata* n* = 121 60%; *p*-value 0.001). Child health (Muhima *n* = 55, 33%; Nyamata *n* = 101 50%; *p*-value < 0.001) and acute care (Muhima *n* = 55, 33%; Nyamata *n* = 97 47%; *p*-value 0.004) were the most common reasons for seeking healthcare. When asked about their willingness to self-collect, 80% (*n* = 135) of women in Muhima and 96% (*n* = 196) of women in Nyamata said yes (*p*-value =  < 0.001).

**Table 1:**
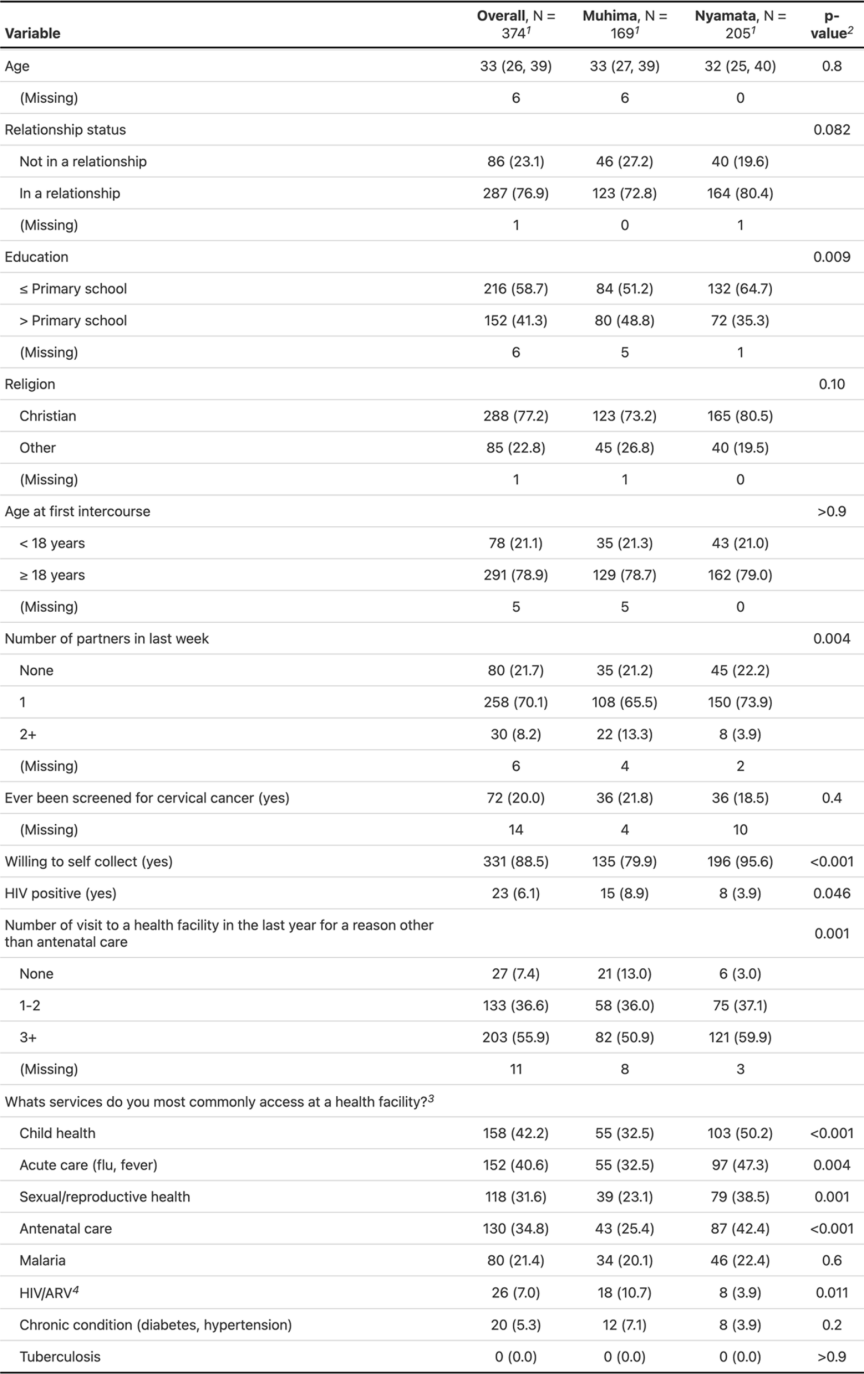
Demographics by site

^1^Median (IQR); *n* (%)

^2^Wilcoxon rank sum test; Pearson’s chi-squared test; Fisher’s exact test

^3^Multiple response

^4^Antiretroviral therapy

Table 2 shows the most common barriers to accessing women’s health services as reported by our surveyed population. Only 9 (5.3%) women in Muhima and 12 (5.9%) in Nyamata indicated no barriers to care (*p*-value 0.8). The majority of women from both Muhima (60.9%) and Nyamata (65.9%) indicated other for their biggest barrier. However, among the listed options, both women in Muhima and Nyamata indicated that long wait times at the facility were the biggest barrier (Muhima *n* = 44, 26%; Nyamata* n* = 55, 27%; *p*-value 0.90). Low quality of care was the second most common barrier (Muhima *n* = 29, 17%; Nyamata *n* = 25, 12%; *p*-value 0.20) followed by transportation costs (Muhima *n* = 22, 13%; Nyamata *n* = 25, 12%; *p*-value 0.80). Women from Nyamata were significantly more likely to report distance to health center as a barrier (Muhima *n* = 7, 4.1%; Nyamata *n* = 32, 16%; *p*-value < 0.001) and women from Muhima were significantly more likely to report the transportation method as a barrier to screening (Muhima *n* = 15, 8.3%; Nyamata *n* = 4, 2.0%; *p*-value = 0.004). Notably, no women reported that “lack of awareness on what services I need” to be a barrier to accessing women’s health services and only one woman from Muhima (0.6%) and three women from Nyamata (1.5%) reported that they had a lack of awareness of where to obtain services (*p*-value = 0.60).

**Table 2:**
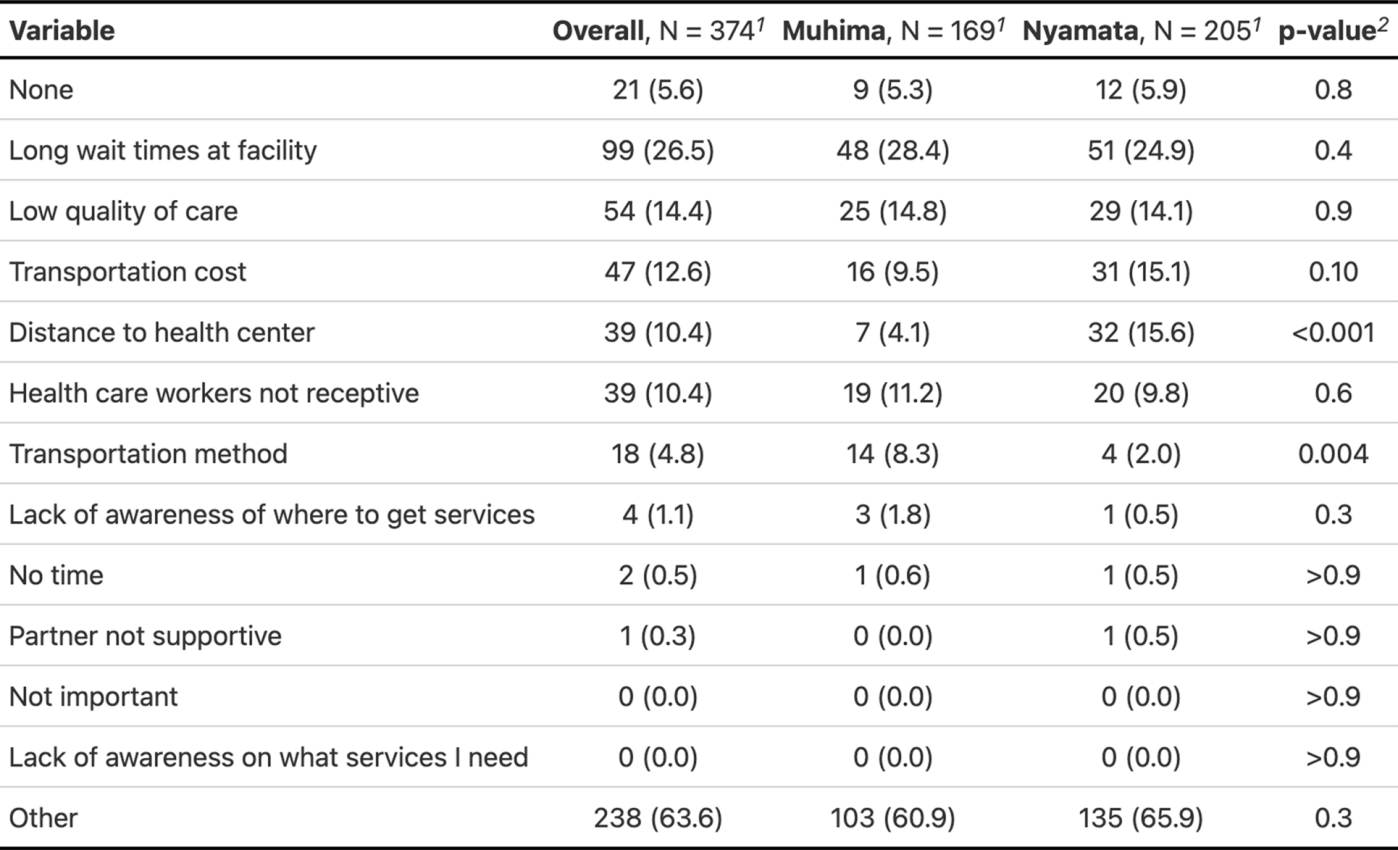
Most common barriers for accessing women’s health services by site

^1^*n* (%)

^2^Pearson’s chi-squared test; Fisher’s exact test

In all, 76% of women from Muhima (*n* = 129) and 78% of women from Nyamata (*n* = 159) had never been screened for cervical cancer (*p* = 0.30). Among these participants, the primary reason for not obtaining screening was that they did not know how or where to get tested (Muhima *n* = 76, 56%; Nyamata *n* = 88, 57%). The least common reasons for not being screened were that the clinic was too far away (Muhima *n* = 1, 0.7%; Nyamata *n* = 2, 1.3%), a family member would not allow it (Muhima* n* = 2, 1.5%; Nyamata *n* = 1, 0.6%), and embarrassment (Muhima *n* = 2, 1.5%; Nyamata* n* = 1, 1.3%).

## Discussion

Participants in this cross-sectional study were recruited from an urban district hospital in Kigali (Muhina, 169, 44.2%), and a rural district hospital in Bugesera (Nyamata, 205, 54.8%). The participants were largely comparable between the two sites. Among both sites, the most common barriers to accessing healthcare services were the quality of clinical care (long wait times at facility, low quality of care, health care workers attitudes) and issues with traveling to the clinic (transportation cost, transportation method, distance to health center). However, women from Nyamata were significantly more likely to report distance to the health center as a barrier and women from Muhima were significantly more likely to report transportation method as a barrier.

The lack of differences in barriers to general health services between the two sites is largely unsurprising. Similar to our findings, long wait times and low quality of care have been reported in previous studies in both urban and rural health centers in Rwanda [12, 24–26]. As of 2019, Rwanda only has 0.1 physicians per 1000 people (in comparison, the United States has 2.6 per 1000 people) [27] and nurses make up the majority of healthcare providers in Rwanda [28, 29]. This human resource shortage may lead to longer wait times and access to limited health services care. It is not surprising that participants from rural regions more often reported distance to the health center as a barrier to screening, research has shown that women in rural areas often travel farther to access health services in LMICs [30, 31]. However, it is notable that women in urban areas were more likely to report mode of transport as an issue; future research should explore access to transport as a barrier to health services in urban Rwanda and how this obstacle could be addressed to improve health outcomes. Additionally, more research is needed to better understand what other barriers women in Rwanda face to obtain cervical cancer screening as the majority of women in both groups indicated “other” as their response.

Among the two sites, the majority of women noted that self-collection would be acceptable for cervical cancer screening. This is important because many of the barriers that women face accessing health services such as low quality of care and long wait times could be eliminated by implementing cervical cancer self-collection screening program in Rwanda. Self-collection in Rwanda could be integrated into primary points of care, such as ANC clinics. Furthermore, self-collection does not require specialized training to perform and would eliminate the need for a pelvic exam. As such, this could lead to improved clinical care as it would allow for community healthcare workers to be trained in implementing the program and would allow women to avoid an unpleasant exam. More research is needed to understand if women would be willing to wait longer if they were to receive multiple services at once when visiting a health center.

The findings from our study largely align with Niyonsenga et al. study on barriers faced by women accessing cervical cancer screening in Rwanda. While our study findings are broader in that they ask women about accessing general health services, the findings from Niyonsenga et al. can be comparable as they reported the most common barriers to cervical cancer screening in Rwanda as lack of information about the importance of screening, availability of services, and wait times. Unlike our study, they did not ask specifically about transportation to clinics. However, they did ask if living in a rural area prevented them from obtaining screening (51.9% agreed) [12]. Similarly, our study findings align with previous research on the acceptability of self-collection for cervical cancer screening. Mitchell et al. conducted a cross-sectional study of 300 women in Kisenyi Uganda reported that 81% were willing to use self-sampling for cervical cancer testing [16]. Esber et al. reported similar results in Malawi [32] and Broquet et al. in Madagascar [33].

This study has several strengths and limitations. First, the study is strengthened by the study team’s knowledge of cervical cancer and past experience conducting a similar study in Uganda [16]. The study is further strengthened by the use of previously used survey instruments. In addition to the strengths, the study has several limitations. First, the study was only conduced at two clinics in Rwanda and thus cannot be considered generalizable to the overall population in Rwanda. Additionally, the study utilized convenience sampling which could lead to selection bias. As the participants were asked questions on a sensitive topic, some women could have altered their responses due to embarrassment, fear, or stigmatization due to the subject matter.

## Conclusions

Our study found that the most common barriers to cervical cancer screening in urban and rural Rwanda were the quality of clinical care and issues with traveling to the clinic. Implementing a cervical cancer self-collection program could help eliminate many barriers found in our study that women face to obtain health services in Rwanda. More research is needed to better understand the acceptability of cervical cancer screening in Rwanda and how it could be integrated into the healthcare system.

## Data Availability

The data used and analyzed during this study includes potentially identifiable personal data. As such, we are unable to make this dataset available in a public depository due to the possibility of identifying participants. Request for data access can be made to Gina Ogilvie at Gina.Ogilvie@bccdc.ca.
